# Successful reconstruction of distal peroneus longus tendon dislocation associated with a split lesion – a case report

**DOI:** 10.1186/s12891-020-03757-6

**Published:** 2020-11-18

**Authors:** Heinz Lohrer

**Affiliations:** 1 European SportsCare Network (ESN), Zentrum für Sportorthopädie, Borsigstrasse 2, 65205 Wiesbaden, Nordenstadt Germany; 2 Department for Sports and Sport Science, Albert-Ludwigs-Universität Freiburg i. Brsg., Schwarzwaldstraße 175, 79117 Freiburg, Germany

**Keywords:** Peroneal tendon dislocation, Peroneal tubercle, Reconstruction, Split lesion, Case report

## Abstract

**Background:**

Peroneal tendon injuries are one of the differential diagnoses in lateral ankle and rearfoot pain. While partial tears are not uncommon, peroneal tendon dislocation at the peroneal tubercle is very rare. Until now, only three papers have been published, presenting five cases of peroneus longus tendon dislocation over the peroneal tubercle. This report adds a previously undescribed case of a peroneus longus tendon split tear that was partially dislocated and entrapped over the peroneal tubercle. The respective operative approach and the outcome are described.

**Case presentation:**

A 25-year-old international top-level speed skater developed a painful mass over the lateral calcaneal wall. There was no specific inducing injury in his medical history. In contrast to previous reports, according to the patient’s history, a snapping phenomenon was not present. Conservative treatment was not effective. By inspection and palpation an enlarged peroneal tubercle was assumed. During operative exploration, we found an incomplete longitudinal split tear of the peroneus longus tendon, which was partially dislocated and entrapped over the peroneal tubercle. This mimicked an enlarged peroneal tubercle. A portion of the split tendon was resected. A deepening procedure of the flat groove of the peroneus longus tendon below the peroneal tubercle and a transosseous reconstruction of the avulsed inferior peroneal retinaculum were performed. After six months, the patient had completely reintegrated into his elite sport and has been free of symptoms since then.

**Conclusions:**

From the presented case it can be speculated that the inferior peroneal retinaculum was overused, worn out, detached, or ruptured due to overpronation and friction the lateral edge of the low-cut speed skating shoe. Then the peroneus longus tendon experienced substantial friction with the peroneal tubercle with possible dislocation during ankle motion. This frictional contact may have finally led to further degeneration and a longitudinal tear of the tendon. Obviously, dislocations can develop insidiously resulting in lesions of the peroneus longus tendon at the peroneal tubercle, ultimately leading to a tendon entrapment. This mimics an enlarged tubercle. The pathology is very rare and can be successfully addressed surgically.

## Background

The peroneal tendons are probably the most important structures in the differential diagnosis of lateral ankle and rearfoot pain [1, 2]. Specifically, peroneal tenosynovitis, longitudinal (split) lesions, and even transverse tears mainly involving the peroneus longus tendon have to be considered [3, 4]. Three distinct zones are used to anatomically locate the pathology of the peroneus longus tendon [5]. Increased shear loads result when the tendon changes its direction at the lateral malleolus (zone A), at the peroneal tubercle (= peroneal trochlea) of the calcaneus (zone B), and in the cuboid notch (zone C). Dislocation of the peroneal tendons generally occurs at the lateral malleolus (zone A) and is the result of an injury to the superior peroneal retinaculum, triggered most frequently by ankle inversion injuries [3, 6–8]. In zone B partial tears are predominantly found, while complete tears occur in zone C [5].

In 2011 another, very rare cause of lateral rearfoot pain was introduced in the literature, resulting from dislocation of the peroneus longus tendon at the peroneal tubercle [9]. Since that case description, only four more cases have been published in two papers to describe this very rare entity [10, 11].

In contrast to tenosynovitis and tears which may be induced by chronic friction of the peroneus longus tendon at an enlarged peroneal tubercle [3, 6], dislocation occurs over normal-sized peroneal tubercles following traumatic tears of the inferior peroneal retinaculum [9–11].

The purpose of this report is to present a previously undescribed case of an incomplete longitudinal split tear of the peroneus longus tendon, which was partially dislocated from its flat groove and entrapped over the peroneal tubercle. This pathology, mimicking an enlarged peroneal tubercle, should be kept in mind by clinicians in the differential diagnosis of lateral ankle and foot pain. The respective anatomic reconstruction addresses all three constituting components of the pathology (inferior peroneal retinaculum, shape of the groove, and peroneus longus split tear). The outcome is assessed by a standardized questionnaire.

## Case presentation

The local Ethics Committee approved the study. Written informed consent was obtained from the patient for publication of this case report and any accompanying images. The rights of the patient were protected. The registration trial number is DRKS00014266 on DRKS. ‘Retrospectively registered’. Date of registration: 06/04/2018.

### History

**Fig. 1:**
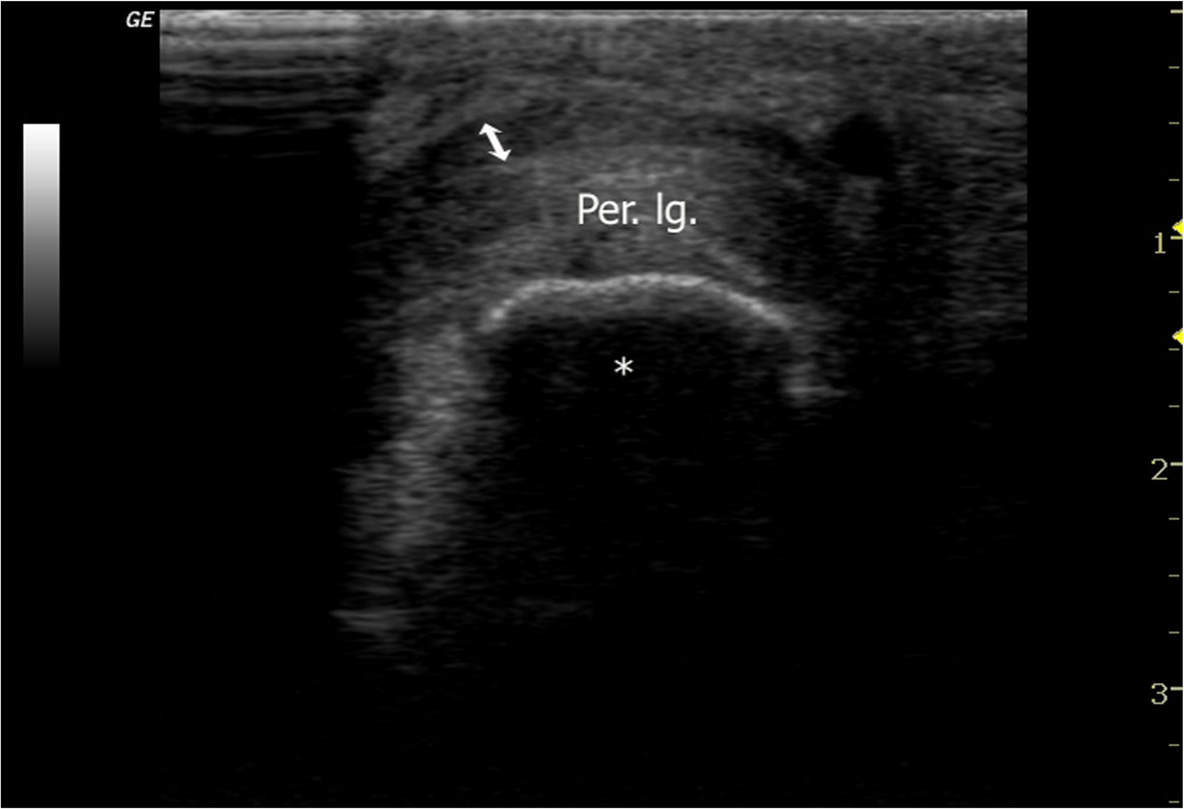
Transverse section ultrasonography of the mass over the peroneal tubercle. An enlarged peroneus longus tendon covers the peroneal tubercle. * = peroneal tubercle. Per. lg. = dislocated peroneus longus tendon. Double arrow = effusion in the peroneus longus tendon sheath

**Fig. 2:**
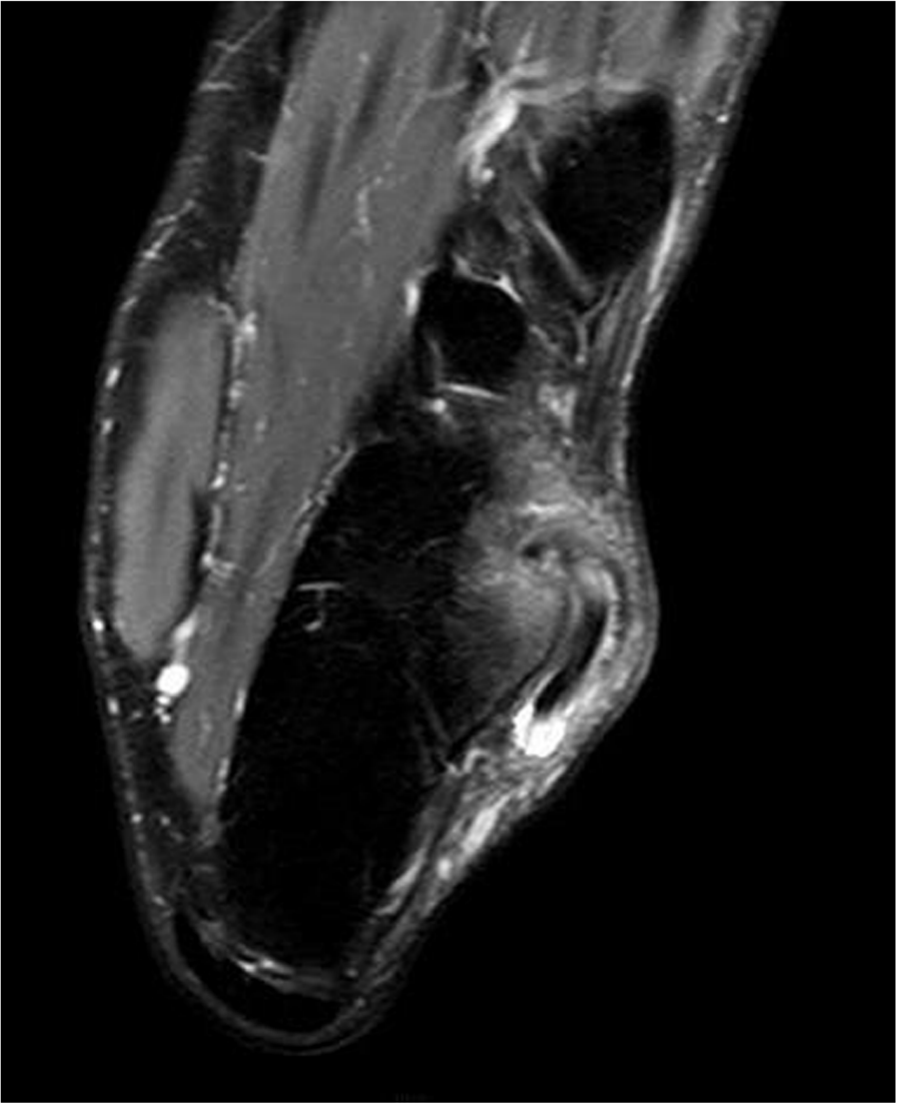
Axial MRI section of the enlarged and dislocated peroneus longus tendon over the peroneal tubercle. Intensive subchondral bone oedema of the peroneal tubercle

**Fig. 3:**
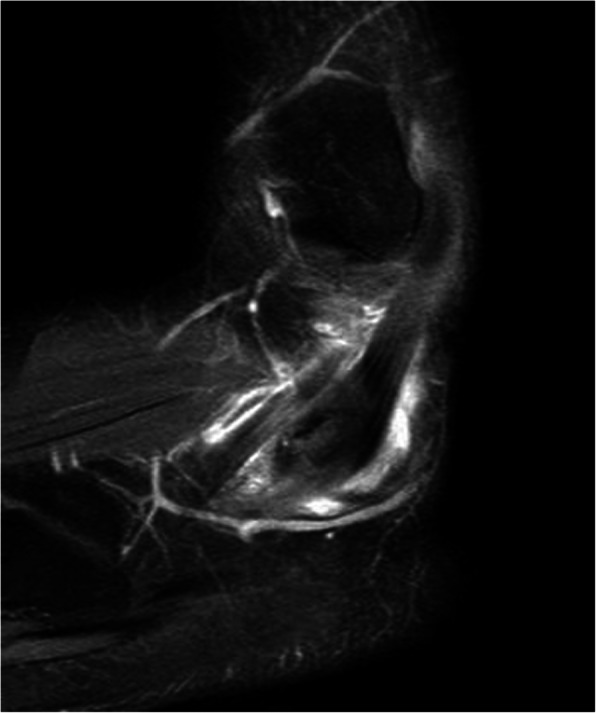
Sagittal MRI section of the enlarged and dislocated peroneus longus tendon over the peroneal tubercle

A 25-year-old speed skater suffered from painful swelling located anterior-inferior to the left lateral malleolar tip. The patient had noticed this mass developing for approximately one year. He reported that he felt no instability and noticed no dislocation or locking phenomenon. A prominence over the lateral calcaneal wall (trochlea peronealis) had already been detected four years ago at a preventive medical check-up, which was performed regularly (once a year), and the prominence had persisted since then. Initially, this prominence was not painful. In these four years, only putting on and taking off a shoe induced minor skin irritation. When he presented himself in the office, he reported a painful mass that developed spontaneously approximately three weeks ago. Pain was induced by speed skating and running. He initially underwent conservative treatment (ice, heat, NSAIDs, and antiphlogistic cream). Examination revealed a hard and tender prominence at the lateral calcaneal wall congruent with an enlarged peroneal tubercle. In addition, physical examination of the foot and ankle revealed normal results. At that time, ultrasound investigation revealed effusion in the inframalleolar peroneal tendon sheath and the peroneus longus tendon located over the calcaneal tubercle (Fig. 1). Two injections (two week interval) into the tendon sheath with 4 mg dexamethasone each substantially reduced the pain, and he was subsequently able to compete. However, the syndrome progressively reduced his ability to practice and to compete in his sport. After three months, MRI (Figs. 2 and 3) verified a peroneus longus tendon split lesion and bone oedema of the peroneal tubercle. The configuration of the peroneal tubercle, based on a recently described CT-classification, was “single-convex” [12]. The height, length, and width measured in MRI were 5 mm, 15 mm and 13 mm, respectively, assuming a normal size of the peroneal tubercle. Operative treatment was suggested, but the patient refused at that time.

### Operative procedure

Outpatient surgery was performed approximately 10 months after the onset of exercise-induced pain because of progressive sports inability despite conservative treatment.

**Fig. 4:**
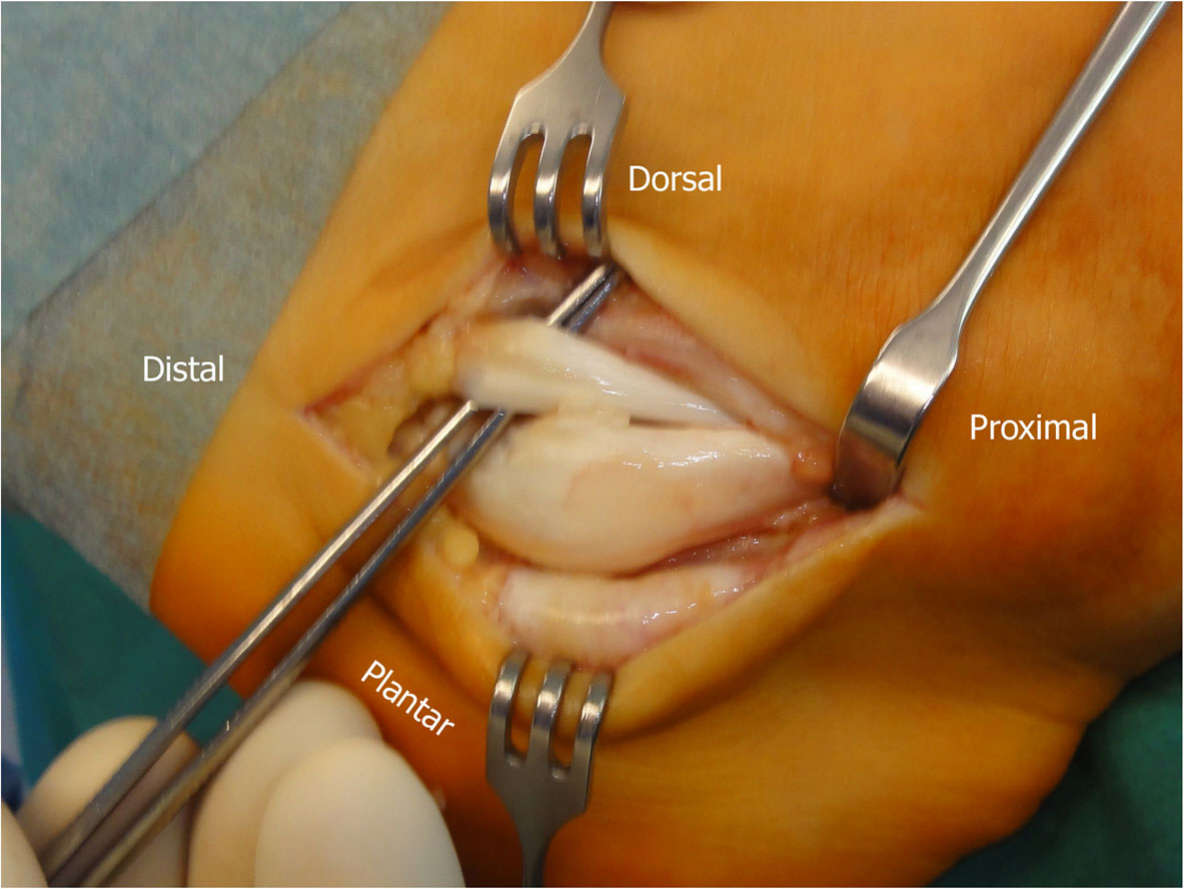
Operative situs. The peroneus brevis tendon (over the forceps) is unremarkable. The peroneus longus tendon is grossly enlarged, frayed, and dislocated over the peroneal tubercle

**Fig. 5:**
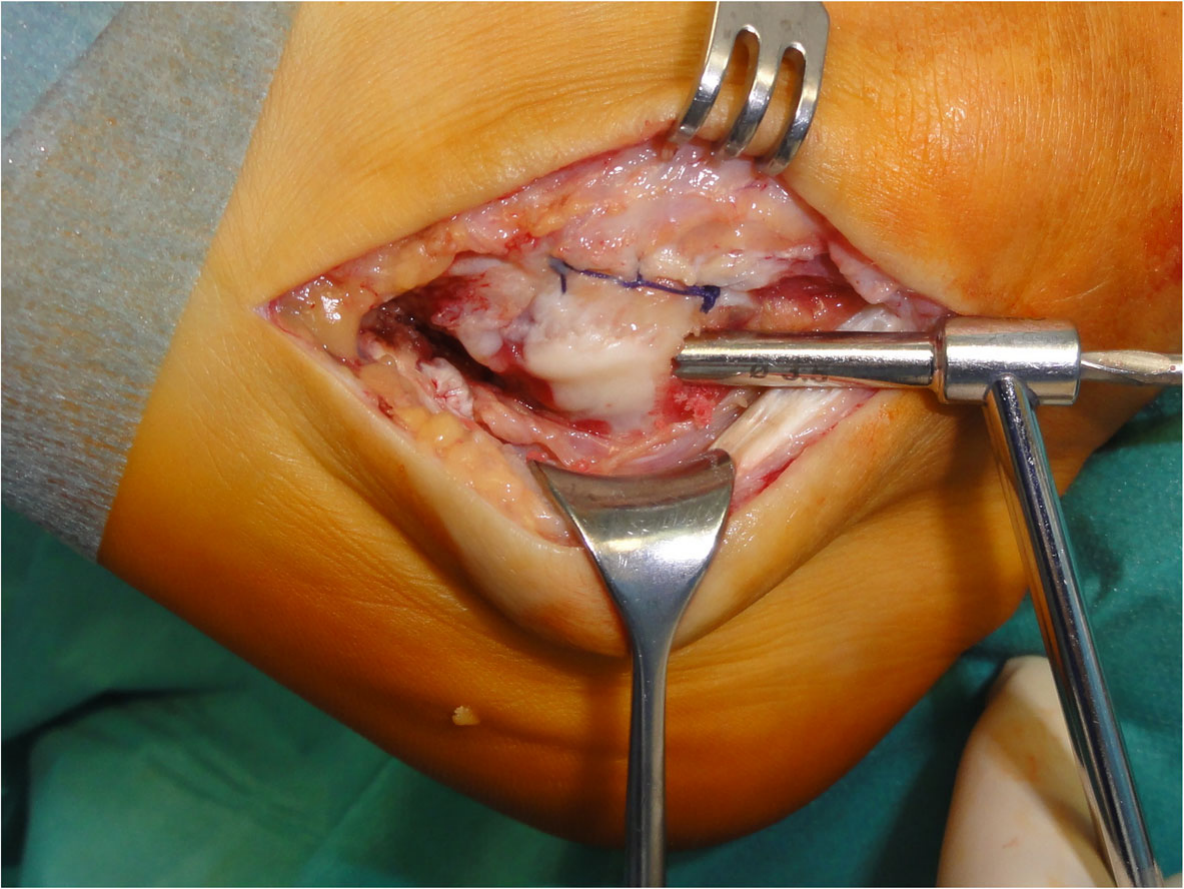
Deepening procedure for the peroneus longus tendon groove below the peroneal tubercle. With the peroneus longus tendon retracted, an oblique subchondral drillhole is made from proximal dorsal to distal plantar. The retinaculum covering the peroneus brevis tendon is already sutured

**Fig. 6:**
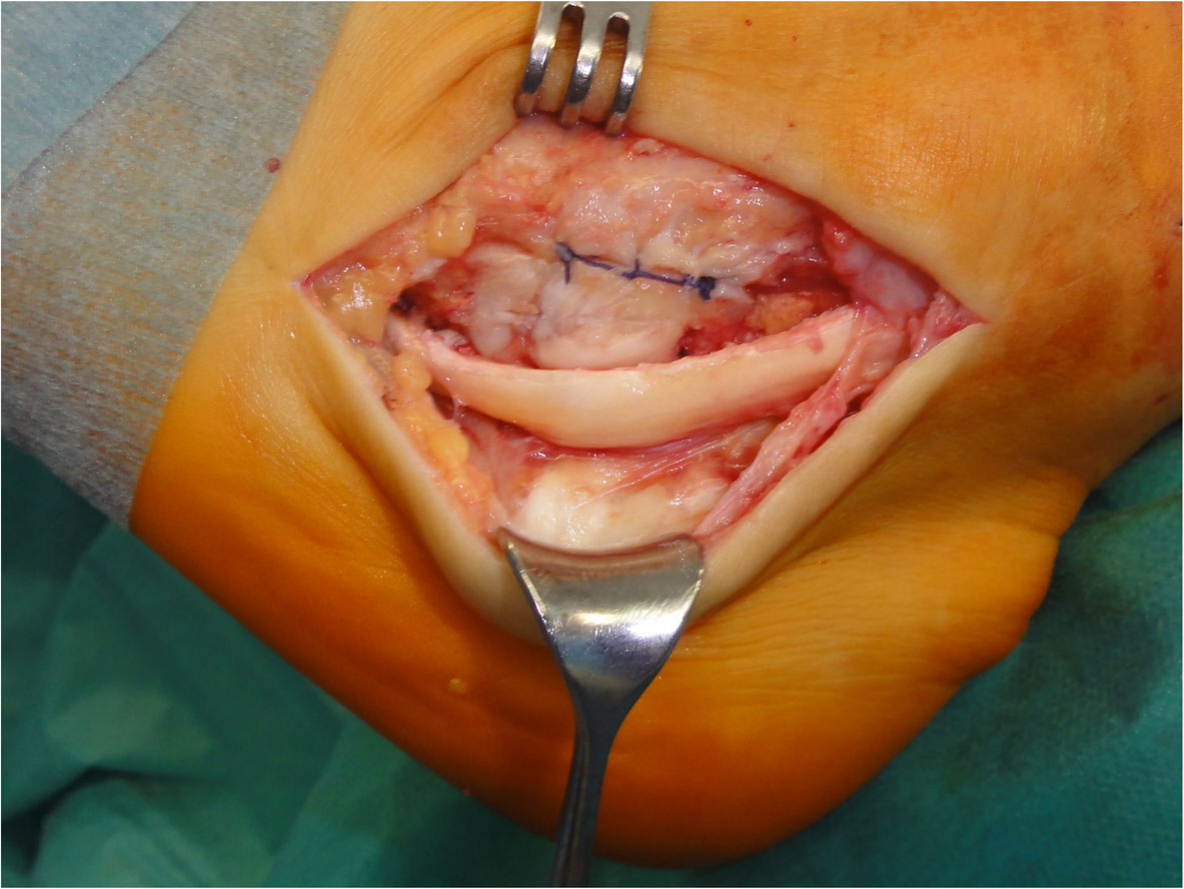
The peroneus longus tendon is repositioned and remains not fully stable in the deepened infra-trochlear groove. The retinaculum covering the peroneus brevis tendon is already sutured

Initially, peroneal tenoscopy confirmed the peroneus longus tendon split lesion and tenosynovitis. A 10 cm curvilinear skin incision was made from the posterior lateral malleolus over the course of the peroneal tendon to the base of the fifth metatarsal. Subcutaneously, a bursa covering the peroneal tubercle was resected. The peroneal tendon sheaths were opened. The peroneus brevis tendon was unremarkable. The peroneus longus tendon was degeneratively enlarged and was dislocated over the peroneal tubercle (Fig. 4). There, the inferior peroneal retinaculum was detached from the peroneal tubercle. Because of this, a pouch was formed, which contained the tendon. The surface of the calcaneal bony groove under the peroneal tubercle was flat. On its calcaneal surface, the peroneus longus tendon exhibited fraying and an incomplete longitudinal tear. Starting from this incomplete split lesion, approximately 50% of the enlarged tendon was resected over a distance of approximately 5 cm. With the peroneus longus tendon retracted, the subchondral bone under the peroneus longus tendon groove was drilled into (2 and 3.2 mm) obliquely from proximal to distal (Fig. 5), and the subchondral bone was further removed. With a tappet rod, the chondral bottom of the groove was impacted. At this point, the repositioned peroneus longus tendon remained not fully stable in its position during passive motion of the foot (Fig. 6).

**Fig. 7:**
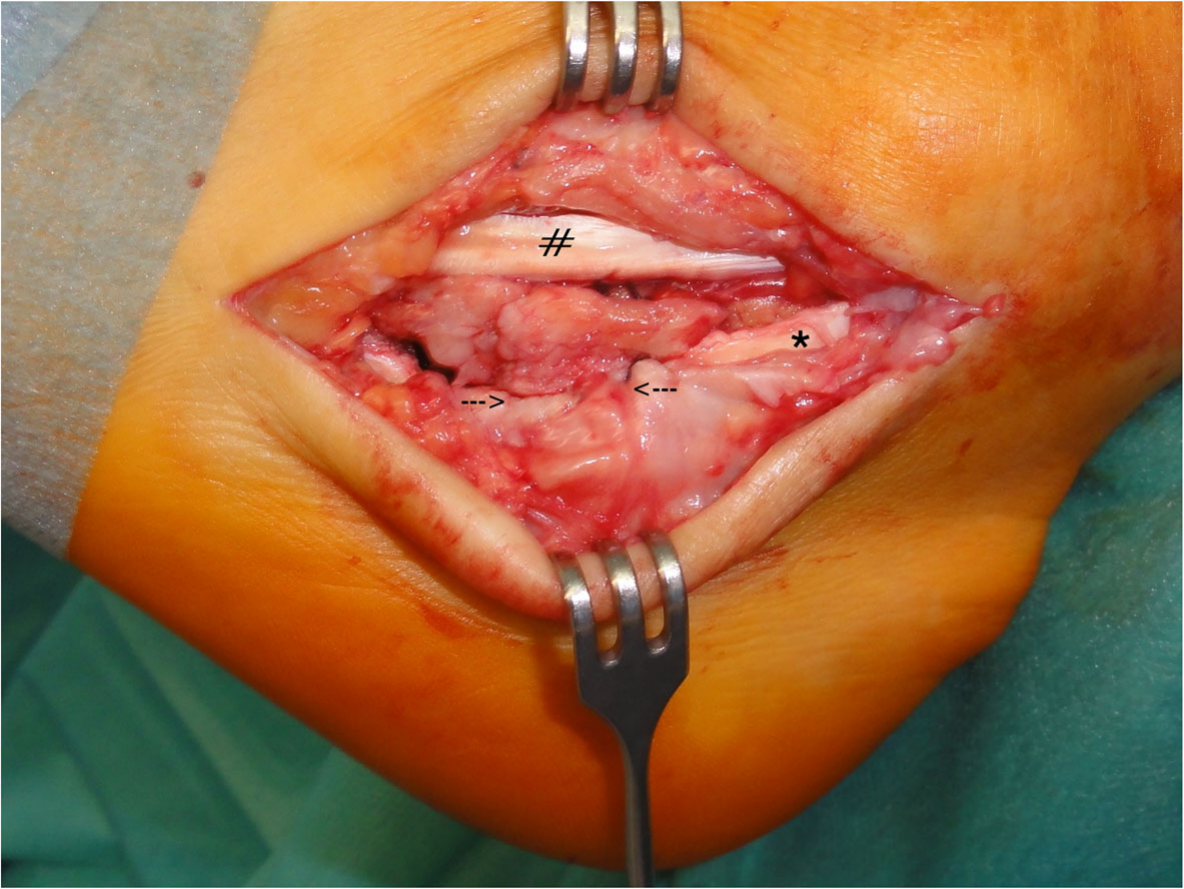
Operative situs after completing transosseous reconstruction of the inferior part of the inferior peroneal retinaculum over the peroneus longus tendon (*) into the peroneal tubercle (between arrows). At this stage, the covering of the peroneus brevis tendon (#) was transiently removed

Because of the flat peroneus longus groove behind the tubercle, a decision was made for additional stabilization by transosseous retinaculum reconstruction. Three parallel 2 mm drillholes were made at the upper edge of the peroneus longus groove tangential into the peroneal tubercle (plantar to dorsal), and a small notch was created at the inferior bony rim using a chisel and rongeur. The enlarged inferior peroneal retinaculum was cut to precisely reach the notch in the peroneal tubercle under moderate tension over the repositioned peroneus longus tendon. Two #0 absorbable sutures were inserted transosseously (dorsal to plantar), and the upper rim of the inferior peroneal retinaculum was stitched with a running suture. The thread was reversed through another drillhole. When tensioning these U-shaped sutures, the inferior peroneal retinaculum was tightened into the notch (Fig. 7). Subcutaneous tissue and skin were closed in a standard fashion. Finally, an elastic compressive dressing and a posterior foot and ankle cast were applied in a slight equinus position.

### Rehabilitation

Postoperatively, the patient wore his cast 24 h per day for one week. The cast then remained for six weeks postoperatively in the form of a night splint. A stable shoe with a 3 cm heel lift was implemented one week postoperatively during the day, and the load was gradually increased over a period of three days. Six and eight weeks postoperatively, full body weight was allowed in a jogging shoe with a 2 cm and 1 cm heel lift, respectively. After the 12th postoperative week no further support was implemented, and the patient started to progressively begin running and speed skating. Physiotherapy was initiated six weeks postoperatively. Six months postoperatively, a full load during practice and competition was resumed.

### Follow-up

Follow-up was performed 2.5 years after surgery. There is no validated instrument to measure the outcome of peroneal tendon injuries. Therefore, the Foot and Ankle Ability Measure – German version (FAAM-G) was chosen as the primary endpoint. The FAAM-G includes activities of daily living (ADL) and a sports subscale. It is “a reliable and valid questionnaire for self-reported assessment of pain and disability in German-speaking patients suffering from chronic ankle instability” [13]. The patient had already completed this questionnaire preoperatively. We defined sports ability in his former sport (speed skating) as a secondary endpoint of the follow-up investigation.

Preoperatively, our speed skater scored 83.3% on the FAAM-G ADL subscale and 18.8% on the FAAM-G sport subscale. The postoperative FAAM-G values increased to 98.8% (ADL subscale) and 95.8% (sport subscale). Preoperatively and at the follow-up, the patient rated the level of function during ‘usual activities of daily living’ to be 90 and 100%, respectively. The overall level of function of the injured foot was assessed as ‘severely abnormal’ preoperatively and ‘normal’ at the follow-up. The level of function during his ‘sports related activities’ was specified at 50% preoperatively and at 98% at the follow-up. Physical examination 1.5 years postoperatively demonstrated unremarkable findings with no swelling or tenderness over the operated area and with full peroneal strength. Sonography revealed a normal peroneal tendon size, echogenicity, and no tenosynovitis. The patient resumed a full speed skating activity level and won the 10 km world championships 28 months after surgery.

## Discussion and conclusions

Previous reports have described either tenosynovitis, tears or, very rarely, dislocation of the peroneal tendon at the lateral calcaneal facet [5, 14, 15]. The case presented in this study is unique regarding its complexity. It combines a split lesion and entrapment of a dislocated peroneus longus tendon at the level of the peroneal tubercle in the midportion of the lateral calcaneal wall. The resulting prominence mimicked an enlarged tubercle. With six published cases, distal peroneus longus tendon dislocation is very rare. From the presented case, it can be speculated that the inferior peroneal retinaculum was overused, worn out, detached, or ruptured due to overpronation and friction of the lateral edge of the low-cut speed skating shoe. Then, the peroneus longus experienced substantial friction with the peroneal tubercle with possible dislocation during ankle motion. This frictional contact may have finally led to further degeneration and a longitudinal tear of the tendon. The initial lesion (detachment or rupture) of the inferior peroneal retinaculum seems not to be associated with tubercle enlargement, which is thought to be a predisposing factor for tendon tears [12, 15]. Intraoperatively, we found a flat peroneus longus groove below the calcaneal tubercle. In the retromalleolar area, a flat or convex groove is thought to be a predisposing anatomic factor for peroneal tendon dislocation [15]. However, there is no research available for objectively assessing the shape of the bony groove at the level of the calcaneal tubercle. Therefore, no conclusions can be made about the pathologic relevance of this finding. In addition, patients with distal peroneal tendon dislocation are younger and are more active in competitive sports than patients suffering from peroneal tendon pathology associated with tubercle enlargement [14]. Additionally, patients with distal peroneus longus tendon dislocation have normal-shaped feet (Table 1). A traumatic induction of the dislocation was not observed in the presented case. Overpronation may have played an inducing role, and one case report demonstrated an ice skater who also complained about an insidious onset of symptoms [10]. Cavovarus feet and enlarged peroneal tubercles seem to predispose patients for lateral ankle sprains and friction pathologies, respectively [3, 5, 14, 16, 17].

**Table 1:** Results of the literature analysis for dislocation of the peroneus longus tendon at the peroneal tubercle

Author(s)	N	Sex [male/female]	Age (years)	Sport	Injury	Additional injuries/anomalies	Peroneal trochlea enlarged	Preoperative History (months)	Symptoms	Snapping	MRI	Conservative treatment	Operative technique	Latest FU (months.)	Result at Latest FU
Lohrer 2020 (presented case	1	1/0	25	Speed skating	No	No	No	10	Lateral foot painful. Soft tissue mass. Skin irritation with shoe	No	PLT split, tubercle edema	Ice, heat, NSAIDs, cream, two cortisone injections	PLT partial resection, PLT groove deepening, IPR reconstruction	30	Excellent
Staresinic et al. 2013 [11]	3	3/0	20 23 28	Soccer	Ankle sprain and prolonged problems on the lateral side of the foot	Ankle sprain, direct blow	No	2.5 1 1	Swelling, hematoma, tenderness around the ankle	Yes	PLT dislocation	Rest, physiotherapy	*tT*ubercle excision, lateral calcanear groove formation for both peroneal tendons and IPR plasty	24	Excellent
El Rassi et al. 2012 [10]	1	0/1	23	Ice skater	No trauma, insidious	No	No	18	Pain on lateral right foot and ankle, sense of instability	Yes	Increased signal at the level of the calcaneal tubercle	Ankle brace, NSAID	IPR reconstructed with part of its superior portion.	36	Excellent
Klos et al.2011 [9]	1	1/0	23	Soccer	Foot caught in plantarflexion, abduction, eversion	No	No	2	Pain and swelling lateral hindfoot	Yes	No associate lesions	NSAIDs, rest, physical therapy, ankle brace	Groove deepening, suture anchor IPR reattachment.	6	Excellent

*FU* follow-up, *PLT* peroneus longus tendon, *PBT* peroneus brevis tendon, *IPR* inferior peroneal retinaculum. Updated from The Journal of Foot and Ankle Surgery, 58, *Heinz* Lohrer H, “Distal Peroneus Longus Dislocation and Pseudohypertrophy of the Peroneal Tubercle: A Systematic Review”, Pages No. 969–973, Copyright (2019), with permission from Elsevier

In contrast with previous reports of distal dislocation of the peroneus longus tendon (Table 1), the presented case is unique because the patient reported no snapping phenomenon at the lateral calcaneal wall. The reason for this is entrapment by the split lesion located over the normal-sized peroneal tubercle, mimicking enlargement of the peroneal tubercle.

Diagnosis of the described condition is made from clinical findings (Table 1). It is important that physicians are aware of this rare lesion.

As demonstrated in this case, imaging (MRI and ultrasonography) can confirm dislocation. However, if a normal-sized tendon is not actually dislocated, imaging may not be helpful. Friction-induced oedema of the lateral calcaneal wall, tenosynovitis, and tendon split lesions are unspecific findings.

All patients with distal peroneal tendon dislocation were unresponsive to conservative treatment (Table 1). Therefore, as a result of the current literature and from the presented case, surgery is recommended as the treatment of choice when conservative treatment fails [15]. The rationale against resection of the whole degenerated peroneus longus tendon and tenodesis to the peroneus brevis is provided by the principle of anatomically restoring anatomy, specifically when considering the demanding loads of top-level athlete. It is expected that the remaining peroneus longus tendon will sufficiently recover. This can be seen analogue in surgery for Achilles tendinopathy, where up to 50% of the tendon can be resected [18]. The assumption is further substantiated ex juvantibus by the excellent result obtained in the described case. The patient completely recovered and was able to compete successfully at the highest international level. He finally assessed the functional capacity of his operated foot as “normal”. When comparing the operative techniques for distal peroneal dislocation within the literature (Table 1), all authors repaired the inferior peroneal retinaculum. Additional groove deepening added stability and safety, specifically for this high-demand athlete.

The strength of the present case report is achieved by the introduction of a groove deepening technique, which provides additional security for inferior peroneal retinaculum reconstruction, and by the prospective evaluation of the patient with the administered outcome measure. It remains unclear whether the excellent result could be replicated with isolated repair, by reinsertion of the inferior peroneal retinaculum, or by groove deepening alone. This question could be answered with further comparative research. A prominent peroneal tubercle can be considered for excision in patients with associated peroneal tendon pathologies [15]. In either published case of distal peroneal dislocation, however, the peroneal tubercle was assessed as “normal” and was not resected. The results of treatment were excellent in all cases (Table 1).

Distal peroneus longus dislocation with a split lesion and entrapment over the peroneal tubercle, is a very rare sports injury. It is difficult to diagnose when the patient does not report tendon snapping. This pathology adds to the differential diagnosis of lateral ankle and rearfoot pain.

In conclusion, detachment of the inferior peroneal retinaculum, peroneus longus tendon dislocation, and entrapment by a split lesion represent a new complex entity that can effectively be reconstructed by partial resection of the split tendon, groove deepening, and transosseous reinsertion of the inferior peroneal retinaculum.

## Data Availability

The datasets used and analysed during the current study are available from the author on reasonable request.

## Abbreviations

FAAM-G: Foot and Ankle Ability Measure – German version
ADL: Activities of daily living
